# Determinants of digital home spirometer use and quality parameters in management of patients with chronic obstructive respiratory disease and asthma in general practice: a mixed methods study

**DOI:** 10.1186/s12913-026-15048-2

**Published:** 2026-06-30

**Authors:** Julia Cummerow, Christina Jana Strobel, Katja Goetz, Jost Steinhäuser

**Affiliations:** Institute of Family Medicine, University of Luebeck, Ratzeburger Allee 160, 23538 Luebeck, Schleswig-Holstein, Germany

**Keywords:** Telemedicine, Chronic obstructive pulmonary disease, Bronchial asthma, Primary care, Chronic disease, Health service research

## Abstract

**Background:**

Determinants of telemedicine applications use by general practice patients with chronic respiratory diseases are not yet fully known. The aim of this study was twofold: (a) to assess facilitators and barriers for the use of digital home spirometers in primary care, and (b) to evaluate parameters for process quality in implementing guideline-recommended care in daily practice.

**Methods:**

The study was evaluated using a mixed-methods design. Patients with bronchial asthma or chronic obstructive pulmonary disease were instructed to take daily measurements using a digital home-spirometry device. Primary care physicians regularly controlled spirometry data using a digital monitoring portal. Telephone interviews were conducted with patients and healthcare professionals. Qualitative analysis of the transcripts was based on content analysis. Quantitative data included the assessment of quality indicators in the beginning (t0) and at the end (t1) of each individual’s participation in the study, the Patient Assessment of Chronic Illness Care Short-Version (t0) and reasons for use/non-use of the spirometer (t1). Logistic regression was performed to determine predictors of use according to instructions.

**Results:**

Altogether, 127 patients participated in the study; the average age was 59 years, 61% (*n* = 78) of participants were female. 21 telephone interviews with patients and healthcare professionals were conducted. Facilitators included: sense of obligation, habituation, visualization of progression, self-efficacy, physician contacts, remote treatment, and positive aspects of integration into daily practice. Barriers included: understanding of the disease, dyspnea, as well as project and technical requirements. Care providers considered time-consuming procedures and clinical assessability as barriers. At t0 and t1, approximately one third of respondents (33% resp. 36%) stated that they had received disease-specific training, and 57% stated in both cases that they had gotten the annual influenza vaccination. Logistic regression revealed that duration of disease (OR 0.96; CI 95% 0.93; 0.99), a daily measurement being too strenuous (OR 0.165; CI 95% 0.04; 0.076), and the fact that measurements could not be taken while away from home (OR 0.235; CI 95% 0.07; 0.75) to be negative predictors of use according to instructions.

**Conclusions:**

For the patients in the study, digital applications improved self-efficacy and self-control. However, a better understanding of these applications and additional medical feedback could be helpful for the patients using such digital applications. The evaluation of process quality parameters showed potential for improving rates of disease-related education and influenza vaccination.

**Supplementary Information:**

The online version contains supplementary material available at 10.1186/s12913-026-15048-2.

## Introduction

Aims of medical care for Chronic Obstructive Pulmonary Disease (COPD) and bronchial asthma are symptom control, preventing progression, and reducing disease-related mortality. Further efforts focus on preventing decline in pulmonary function and on providing advice regarding indicated vaccinations, as well as smoking cessation. Individualized pharmacological treatment regimen flanked by improved self-management and education are additional goals [1, 2]. Hence, concepts including these components are needed to provide comprehensive medical care.

One concept for structured care for patients with chronical illness in general is the Chronic Care Model (CCM). It aims to provide proactive routine care and improve the patients’ understanding of their disease [3]. In Germany, the concept of CCM was partly introduced in 2002 through Disease Management Programs (DMPs), also enabling structured outpatient care for patients with asthma or COPD [4, 5].

Although some positive effects have been reported, e.g. regarding adherence to guidelines and therapy [6, 7], the program is rather inflexible when it comes to defining the type and frequency of recording disease-specific parameters [5].

A better understanding of inflammatory processes and biomarker influence provide an important basis for a more personalized care, which is considered to be necessary for overcoming a fixed “one size fits all” management [8, 9]. The guidelines also call for long-term health care that involves a patient-provider partnership and promotes self-management and empowerment [1, 2].

Newer technologies of telehealth including home spirometry can be considered for this purpose. While initial results appeared promising with positive effects on treatment costs, emergency hospitalization rates, duration of hospital stays, and mortality [10], a three-year evaluation eventually showed increased treatment costs, more drug prescriptions, and higher rates of outpatient contacts. On the other hand, the survival rates improved [11]. A higher quality of life for COPD patients receiving telemedical care was reported in comparison to a non-intervention group. However, no difference was found regarding the severity of disease-related symptoms [12]. A Cochrane review found no evidence that telemedicine was associated with less disease progression, improved quality of life, fewer symptoms of respiratory distress, hospitalizations, or death. There was some evidence of a small reduction in hospital readmissions when telehealth was used in combination with usual care [13]. Thus, the impact of telehealth on the care for patients with COPD or asthma is not yet fully understood.

In Germany, there is a high density of doctors, especially in urban regions, while rural regions require more extensive measures to ensure close-to-home-care [14]. Telemedicine approaches are considered to facilitate pneumological care where long distances and long waiting times must be accepted in order to have access to a pneumologist [15]. Nevertheless, further analysis is needed to better understand the barriers and facilitators of telehealth in the medical care for patients with COPD or asthma.

The aim of this mixed-methods study was twofold: (a) to qualitatively evaluate barriers and facilitators to patient use of a digital spirometer and monitoring thereof by primary care providers, and (b) to quantitatively assess the implementation of guideline recommendations using selected patient care quality indicators in daily practice.

## Methods

A mixed-methods study design as well as elements of convergent parallel design and an exploratory mixed-methods design were used [16]. The Consolidated Criteria for Reporting Qualitative Research (COREQ) for collected qualitative data and the checklist Strengthening the Reporting of Observational Studies in Epidemiology (STROBE) for collected quantitative data were followed [22, 23].

### Project information

The project Telemedical Pulmonary Function App with Networking (TeLAV) was conducted in the region of Rendsburg, northern Germany, from April 2021 to March 2024. Patients with asthma or COPD were provided Nuvoair’s AirNext^®^ digital home spirometer with an app for one year. The attending physicians were provided with a digital portal that allowed asynchronous monitoring of data transmitted via Bluetooth^®^. The spirometer AirNext^®^ and the App are Class IIa medical devices; the digital physician portal is a Class 1 M medical device. The spirometer has been validated for different pulmonary conditions [17].

Patients were instructed to record and transmit lung function data at least once a day. Physicians were asked to monitor this data twice a week and at any additional time considered necessary. The project’s pneumological assistant provided individual training for patients and practice staff on all digital devices. No further intervention beyond standard care was planned.

### Qualitative study

In order to determine barriers and facilitators for the use of digital devices as well as their practicability and usability, semi-structured telephone interviews were conducted. The interview guide was based on previous work and the authors’ experiences (JC, internal medicine, MPH, CS, medical student, JS, general practitioner and health service researcher), and was led by an experienced qualitative researcher (JS). The comprehensibility of the interview guide was tested by means of two trial interviews, after which feedback was given to the interviewer. No changes needed to be made to and the results of the test interviews were incorporated into the analysis. The final interview guide included introductory and additional questions tailored to the background of the respective participant.

The questions did not have to be asked in the given order and it was possible to follow up or rephrase questions as needed. The interview guide questions referred to in this study are reported in Supplementary File 1.

### Quantitative study

In the beginning (t0) and at the end (t1) of their participation, patients were asked to complete an evaluation form. At t0 and t1 the questionnaire contained sociodemographic information and questions derived from the Quality Indicators for Outpatient Care of Patients with Chronic Lung Disease [18]. Responses were given using dichotomous yes/no options, except for one question on number of hospital admissions (free text). Furthermore, participants answered the “Patient Assessment of Chronic Illness Care (PACIC) Short-Version” [19] at t0, which is used to assess the quality and patient-centeredness of care for people with chronic illnesses [20]. The short version has been shown to have good convergent construct validity and high internal consistency (Cronbach’s alpha 0.87) [19]. Responses to the eleven questions in this patient assessment can be given on an eleven-point percentage scale (in 10% increments between ‘never 0%’ and ‘always 100%’). However, the PACIC items three, six and eleven were slightly adapted for this specific study population. Patient satisfaction with medical care within the last six months was asked in the same way at t0, and in a single free-text question at t1. Based on the results of the qualitative analysis, the t1-questionnaire consisted of twelve questions about reasons for use or non-use (given as table checkbox option) and participants’ perception of the usefulness of their participation in the study. In addition, sociodemographic characteristics of participating practices, including their staff, were also collected.

A translation of the parts of the questionnaire referred to in this study are presented in Supplementary File 1.

### Study participants

The option to participate in this study was offered to all practices in the Rendsburg Medical Quality Network, an association of regional physicians with the aim of improving professional cooperation and patient care. The participating practices were responsible for patient recruitment. Patients were recruited between November 2021 and September 2023. After submitting signed written informed consent, patients were included in the study. Inclusion criteria were a diagnosis of COPD or bronchial asthma and possession of a smartphone. The exclusion criterion was withdrawal of consent. Patients were also excluded if they transferred to a non-participating primary care physician, moved outside the project area, or were admitted to a long-term inpatient care facility.

Participants for the qualitative part of the study were recruited from the quantitative study population. Over time, 26 patients and staff from 13 of the 15 participating practices were invited by email or post to participate in semi-structured telephone interviews. Eligibility for these telephone interviews required at least two months of project participation at the time of the interview. In addition, participants were also included in the quantitative part of the study.

### Data analysis

#### Qualitative data analysis

The interviews were conducted by one researcher (JC) via telephone between March 2022 and August 2023. No third parties were involved. No participant was personally known to the interviewer. Information about the interviewer’s background and the purpose of the study was provided in the written consent form and by telephone before to the interview. Field notes were taken. Interviews were digitally recorded, pseudonymized, and subsequently transcribed verbatim by the first author (JC) and research assistants. Transcription software f4 was partially used. Any spoken information that could be used to identify individuals was pseudonymized. Recruitment was terminated when data saturation was reached (i.e., no additional major themes were emerging [21]). In addition, no more staff members from the participating practices were willing to take part in a telephone interview.

Two researchers (JC, CS) performed the structured qualitative content analysis according to Mayring case by case [22]. The researchers independently identified deductive categories based on the interview guide followed by the supplement of inductive categories and subcategories. Representative anchor quotes were defined for each subcategory. The entire analysis process was discussed with and supervised by a researcher experienced in the methodology (JS) to develop a final consensus version of the identified codes.

#### Quantitative data analysis

Quantitative data were analyzed using IBM SPSS Statistics for Windows, Version 29.0.1.1. Descriptive statistics included means for metric data as well as frequencies and percentages for categorical data. Inferential statistics included a non-parametric sign test to assess differences in qualitative indicators between the beginning and the end of project participation. Medians and a Wilcoxon signed-rank test were used to detect differences in central tendencies of satisfaction with medical care in the last six months at t0 and t1. Based on the documented total number of measurements and the months of participation, a dichotomous variable “*adherence to instructions”* was created (yes = 1, no = 0). A cut-off value of 200 measurements was applied for participants with twelve months of participation. Owing to the foreseeable conclusion of the project in March 2024, the planned duration of participation was shortened as of April 2023, but remained at a minimum of six months (the cut-off value was adjusted accordingly). The target was considered unmet if the cut-off value was not reached and/or the planned participation period was terminated prematurely.

To identify factors that influence the dichotomous variable “*adherence to instructions*,” Spearman’s rho correlations were used to analyze the socio-demographic and quality indicator variables, satisfaction with chronic disease care, and variables concerning reasons for use or non-use. Variables with statistically significant correlations were entered in a binary logistic regression analysis with the dependent variable “adherence *to instructions*”, which is a common method to reduce the number of variables for the logistic regression analysis. The goodness of fit of the logistic regression model was assessed using the Hosmer-Lemeshow test [23]. Effect sizes of statistically significant predictors in the regression model were determined on the basis of the standardized beta (β) coefficient with values > 0.5 indicating a strong effect, values between 0.2 and 0.5 a moderate and < 0.2 a weak effect [24]. In the case at hand, statistical tests with a significance level of 0.05 were used.

#### Ethical approval

Ethical approval was obtained from the ethics committee of the University of Lübeck (approval number 17–224), and the study was conducted in accordance with the Declaration of Helsinki. Each participant provided written informed consent.

## Results

### Sociodemographic information

A total of 15 practices and 127 patients with either COPD or asthma participated in the project. Of the 127 patients, 61% were female participants. The diagnosis of bronchial asthma was more common than COPD (58% vs. 38%). The patients’ mean age was 58.7 (SD 14.8) years and the mean disease duration was 15.3 (SD 14.9) years. For further details on sociodemographic data and practice information, see Tables 1 and 2.

**Table 1:** Sociodemographic information of participants

	Patients included in quantitative study (*N* = 127)		Participants included in qualitative study (*n* = 21)	
	*n* (%)	M (SD), range	*n* (%)	M (SD), range
Female	78 (61)		9 (43)	
Male	48 (38)		12 (57)	
Patients	127 (100)		16 (76)	
COPD	48 (38)		6 (29)	
Asthma	73 (58)		10 (48)	
General practitioner			3 (14)	
Medical assistant			2 (10)	
Mean age (years) (SD), range		58.7 (14.8), 18–82		57 (15.9), 23–82
Disease duration (years)		15.3 (14.9), 0.8–74.0		

Note. M = Mean, SD = standard deviation

**Table 2:** Practice information (*N* = 15)

Participating practices	*n* (%)	M (SD), range
Urban working place	6 (40)	
Rural working place	9 (60)	
Number of doctors with full-time jobs		2.4 (1.5), 1–6
Number of Medical assistants with full-time job		3.2 (1.9), 1–6
Participating doctors	15 (100)	
Female	5 (33)	
Male	10 (67)	
Mean age (years)		52.3 (10.3), 33–66

Note. M = Mean, SD = standard deviation

### Qualitative results

A total of 16 patients and five healthcare professionals were recruited for telephone interviews. Thus, response rates for patients and medical professionals were 61% and 26%, respectively. Nine participants (43%) were female. Table 1 also provides further sociodemographic information.

The average interview duration was 10 min (range: 4–26 min). Identified facilitators and barriers to the use of the digital devices are presented in Supplementary File Table 1, together with corresponding anchor quotes. Where applicable, additional quotes are provided in the text below.

### Facilitators

The regular use of the spirometer was fostered by the individual participant’s perception of obligation to fulfil the project’s requirements. This was further supported by becoming familiar with regularly performing the measurements.

One advantage reported by patients was the visualization of lung function, which allowed for self-monitoring. Some participants reported an enhanced sense of security through this self-monitoring, which was reinforced by the doctors’ external monitoring. Furthermore, patients reported that they correlated the visualized spirometry results with their self-perceived condition. This, in turn could lead to an increase in knowledge and ultimately self-efficacy. Another key aspect of increased self-efficacy was self-management, including the use of medication, and the ability to respond earlier to signs of deterioration. These benefits were also highlighted by doctors:

Then at some point, it also has, how should I put it, a very clear educational character, so you can really use it as feedback system. We once had a patient who, I think, forgot to take his sprays regularly and then realized thanks to the spirometry: ’oh I’m getting really bad so I just have to take care of it more consistently.’ Or if I get an infection and get worse, I immediately notice that the values are worsening. So, there is a feedback component that keeps the patient on the system. (TN13 GP)

For some participants, regular feedback led to a perceived strengthening of their interaction with the doctor. Contact frequency as well as disease-related conversations, for example, were intensified. From a patient’s perspective, the concept thus even improved GP care compared with pneumological care.

One of the identified positive aspects of remote treatment was the reduced number of practice visits and in-practice spirometry. The application enabled proactive medical intervention when signs of deterioration were detected.

From the medical staff’s perspective, the integration into daily practice routine was facilitated by providing a tool for asynchronous monitoring, allowing the physicians to analyze their patients’ spirometry data whenever it was convenient. In addition, the efficiency of being able to simultaneously monitor several patients was highlighted. Furthermore, providing access to the entire team enabled use by all members of the medical staff.

Well, the fact that, with a relatively simple click, I can get an overview of the clinical parameters of these patients, that’s simply an advantage, yes, and also that I can then intervene accordingly, if necessary, i.e., call them back. (PT14 GP)

### Barriers

On the other hand, several barriers were also mentioned. Some patients did not perceive any change in the management of their disease. Some patients mentioned difficulties in understanding the measured values. Also, a lack of medical feedback was reported, not only in this context. One patient, for example, felt unable to draw conclusions from the measurements by himself and without placing them in his specific medical context.

What makes me so…, I missed a clear strategy behind it. I was constantly measuring, but I did not understand the strategy behind it, what I was actually doing (PT11)

One limitation of the telemedical application itself was the lack of feasibility in cases of severe dyspnea. Other barriers were related to project expectations, such as daily measurements. Some participants reported that they forgot measurements only on some occasions, others reported to have forgotten them more often. When there were changes in daily routines, during vacation or on weekends for example, the daily measurements were more challenging for some participants.

For both, medical staff and patients, technical requirements such as the need for a newer smartphone as well as the need for an internet connection and activated Bluetooth were barriers. Regarding project recruitment, it was assumed that some older patients would be overwhelmed by the technological requirements and were thus not invited to participate.

Some physicians reported that they missed clinical details such as facial expressions and nonverbal clues in their medical assessment.

Well, this is not really a disadvantage, I just don’t see the patient. I don’t have a clinical view for it, I just look at the measurement parameters in the first place, where I then of course apply it, and if, because I know the patient, you can also judge it differently, but that’s just the way it is, I say, the nonverbal clues always play a role in everything, and I don’t get that represented in that sense. (PT14 GP)

Physicians also mentioned difficulties with regard to delegating tasks to their practice staff, because they estimated their regular workload too high for any additional duties. The training of patients and the installation of the systems originally carried out by an external project assistant, was found to be too time-consuming for implementation in the practice.

### Quantitative results

The response rate at t0 was 100%. At t1, responses to questions concerning quality indicators were returned by 90 participants (response rate 71%) and responses to questions concerning reasons for use/non-use were given by 85 participants (response rate 67%).

The average duration of participation was nine months, with an average of 20 measurements per month (target: approximately 30). Project requirements for measurement frequency were met by 43% of participants. During the course of the project, seven people died with no relation to their participation in the project.

Table 3 presents the relevant results of surveyed quality indicators. There were no significant changes between t0 and t1.

**Table 3:** Qualitative indicators at t0 and t1, non-parametric sign test

	t0 yes (%) (*N* = 127)	t1 yes (%) (*N* = 90)		Significance of change t0/t1 *p*-value
Disease related education received	42 (33)*	32 (36)	negative differences 5, positive differences 6, bonds 77	1.0
Nicotine abuse	36 (28)	19 (21)	negative differences 4, positive differences 0, bonds 84	0.125
Of these Tobacco cessation advice received	7 (19)	6 (12)	negative differences 2, positive differences 3, bonds 2	
annually Influenza vaccination	72 (57)	51 (57)*	negative differences 4, positive differences 5, bonds 79	1.0

Note. *One missing value

**Fig. 1:**
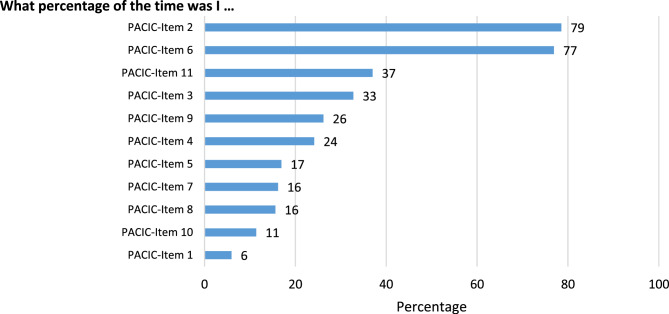
Perceived implementation of chronic disease management aspects in the last six months (*N* = 123). PACIC- Item 1…given choices about treatment to think about. PACIC- Item 2 …satisfied that my care was well organized. PACIC- Item 3 …helped to set specific goals for a healthier lifestyle (e.g. healthy nutrition, exercise). PACIC- Item 4 …given a copy of my treatment plan. PACIC- Item 5 …encouraged to get to a specific group or class to help me cope with my chronic condition. PACIC- Item 6 …asked questions about my health habits (e.g. whether I smoke). PACIC- Item 7 …helped to make a treatment plan that I could carry out in my daily life. PACIC- Item 8 …helped to plan ahead so I could take care of my condition even in hard times. PACIC- Item 9 …asked how my chronic conditions affect my life. PACIC- Item 10 …contacted after a visit to see how things were going. PACIC- Item 11 …told how my visits with other types of doctors, (e.g. a referral to an eye doctor), helped my treatment

The results of the PACIC short version indicated that the highest ranking items were “*satisfied that my care was well organized”*, “*asked questions about my health habits (e.g. whether I smoke)”*, and *“told how my visits with other types of doctors (e.g. a referral to an eye doctor) helped my treatment”* (Fig. 1), whereas “*helped to plan ahead so I could take care of my condition even in hard times”*, “*contacted after a visit to see how things were going”*, and “*given choices about treatment to think about”* showed the lowest mean values.

The Wilcoxon test did not reveal statistically significant differences in patient satisfaction with medical care over the last six months between t0 (*Median* = 80.0) and t1 (*Median* = 83.5) (z= -0.577, *p*= .564, *n* = 80).

**Fig. 2:**
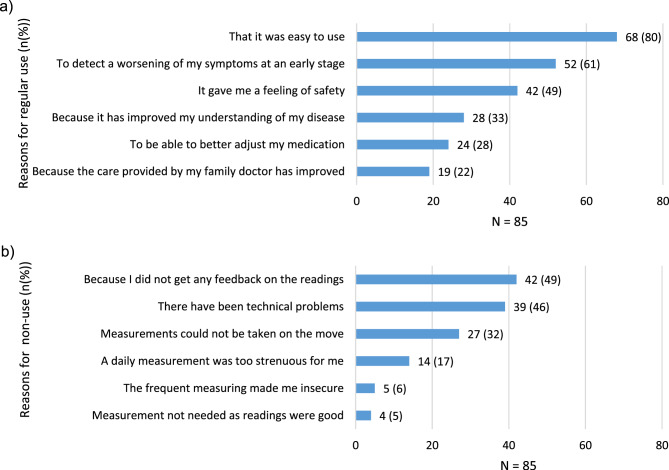
Reasons for regular use/non-use (*n* (%))

At t1, the analysis showed that 87% of respondents considered their participation in the project useful in retrospect. The most common reasons for regular use of the spirometer were “*that it was easy to use*” (80%), “*to detect a worsening of my symptoms at an early* stage (61%), and *“gave me a feeling of safety* (49%). The most common reasons for not using the spirometer regularly were “*because I did not get any feedback on the readings”* (49%), *“technical problems”* (46%), and “*measurements could not be taken on the move”* (32%) (Fig. 2).

Logistic regression (Table 4) revealed that “*duration of disease”*, “*daily measurement was too strenuous”* and *“measurements could not be taken on the move”* were negative predictors of *“adherence to instructions”*, with “*daily measurement was too strenuous”* and *“measurements could not be taken on the move”* indicating strong effects and “*duration of disease”* a medium effect. The model accounted for 30% of the variance. (Nagelkerkes R² = 0.30, Hosmer-Lemeshow-Test *p* = .322). While “*duration of disease”* was a significant predictor, “*age”* was not.

**Table 4:** Logistic regression model, target variable *Adherence to instructions* (*N* = 79)

	β	OR (95% CI)	*p*-value
Gender	0.432	1.540 (0.50; 4.74)	0.452
Age at t0	0.042	1.043 (1.0; 1.09)	0.054
Living alone	− 0.306	0.736 (0.2; 2.65)	0.640
Duration of disease	− 0.041	0.960 (0.93; 0.99)	0.029
A daily measurement was too strenuous for me	-1.804	0.165 (0.04; 0.76)	0.021
Measurements could not be taken on the move	-1.448	0.235 (0.07; 0.75)	0.015
Disease related education received at t1	0.708	2.030 (0.68; 6.03)	0.203
Constant	-1.969	0.140	0.254

Note. OR = odds ratio; 95% CI = 95% Confidence Interval; β = regression coefficient

## Discussion

The purpose of this mixed-methods study was to identify the determinants of digital spirometer use by patients and digital monitoring by primary care providers as well as parameters of quality in patient care. Identified facilitators for spirometer use included improved self-efficacy and benefits of external and self-control from the patients’ perspective. However, some patients also reported a lack of knowledge gain and medical feedback. Physicians appreciated the efficient monitoring and the ability to detect deteriorations early on. However, they were skeptical that the project would be feasible without an external study nurse, particularly given the time-consuming process of familiarizing patients with the equipment. “*Duration of disease”*, *“daily measurement was too strenuous”*, and *“measurements could not be taken on the move”* proved to be negative predictors of *“adherence to instructions”*.

Qualitative data showed that some patients’ perception of disease control changed. The combination of external monitoring and self-monitoring seemed to account for much of the perceived advantage and promoted a sense of security. This corresponds to the finding of other researchers, who report a “sense of security and control” and “better self-management through regular self-monitoring and demand-oriented medical feedback” [25, 26]. Improvements in patient empowerment to better manage chronic lung disease, including medication use and earlier responses to lung function decline, were also reported. This is in line with the asthma and COPD guidelines, which recommend measures to improve self-management and education [1, 2]. Our findings suggest that the changes go beyond improved self-management to include enhanced self-efficacy. Nevertheless, quantitative evidence for such an association is scarce. An overview of systematic reviews regarding use of telemedicine by older patients with various chronic conditions showed that self-efficacy, patient’s active participation, motivation, and mastery to be positively influenced by increased self-care and self-monitoring. However, it was emphasized that self-efficacy has rarely been tested as an outcome variable and that factors influencing the process from use of telemedicine to increased self-management and self-efficacy are considered to be insufficiently researched [27]. Nevertheless, the perceived satisfaction with medical care in this study was high and there was no difference to be observed before and after the project participation.

Quantitative data also showed that medical care was perceived as well organized and that inquiries about health habits were also conducted regularly in daily practice. On the other hand, help in developing a plan to manage the disease during difficult times, practical questions about the state of health, and the presentation of alternative treatment options appeared to be less frequent parts of the medical consultations and should be included more frequently. However, these parameters were only determined at t0 in this study, so it is unclear whether changes occurred during the period of spirometer use. This should be investigated in future studies. There were no statistically significant changes in the parameters measured before and after the period of spirometer use. Disease-related education suggests room for improvement, as only one third of respondents reported to have received such education. Guideline recommendations therefore do not appear to be adequately met. This is consistent with a study that, among others, found significant quality deficits in documented education programs, assessment of smoking status, inhaler technique, and influenza vaccination among COPD patients in Germany [28]. The vaccination rate detected in this present study (57% at t0 and t1) was higher than in the above-mentioned study and the reference for adults with chronic disease in the corresponding region (42%) [29] It does, however, still not meet the target of 75% coverage set by the WHO [30]. According to GPs, reasons for refusing a recommended vaccination include fear of side effects, not perceiving influenza as a threat, general anti-vaccination attitudes, doubts about the effectiveness of vaccinations, as well as positive attitudes towards alternative medicine [31]. This should be taken into account when giving vaccination advice in order to further improve vaccination rates.

Because of the perceived lack of medical feedback, one participant found it hard to independently draw conclusions from the measurements. The most common reason for not using a spirometer regularly was, in fact, not receiving medical feedback. This gives rise to the hypothesis that the benefits of telemedicine applications for COPD treatment depend on human interpretation and interaction, at least for some people. Results of an RCT, in which person-centered telephone support stabilized or even improved self-efficacy in patients with heart failure and/or COPD compared with a control group, support this hypothesis [32]. A medical assistance program designed to improve self-management among patients with chronic diseases showed inconsistent results in terms of hospitalizations for COPD after one and two years, but significantly improved quality of life and overall health after two years [33]. According to our results, external monitoring and more frequent physician-patient contact were also perceived as facilitators. However, the role of physician feedback and external monitoring should be the subject of further research. Nevertheless, the PACIC short version showed no statistically significant difference in perceived satisfaction with medical care before and after the period of spirometer use.

Some participants in our study reported no impact on their dealing with their illness and difficulties in understanding measurements. There was some evidence that certain personal traits are associated with patients’ satisfaction in other fields of telemedicine such as telerehabilitation [34]. Similar influences may therefore have been present in this study and may represent a useful approach for future research to identify those who could benefit most from such a method. In terms of long-term implementation, barriers such as the technical requirements of a smartphone and internet/Bluetooth connection seem to be relatively easy to overcome. Others, such as the lack of feasibility in case of dyspnea and regular daily measurements could be more difficult to address. However, once patients and doctors are no longer bound by study requirements, telemedical monitoring and contact could be more flexibly adapted to the individual patient needs. Good physician-patient communication about these points seems to be necessary. If this is the case, monitoring technologies, along with careful consideration of inflammatory processes and biomarker influences, could play an important role in more personalized care [8].

In addition, fewer practice visits could save time in a context of increasing work intensification and medical staff shortages. These savings in time could help overcome the barrier of time required for patient education and delegation to practice staff, which was perceived as additional work. Although skepticism was expressed about the limitations regarding clinical impression, the possibility of a proactive early response to deteriorating spirometry results may outweigh this and may provide a strong argument for long-term use of the technology in question in primary care.

Logistic regression revealed that those who indicated that “*a daily measurement is too strenuous”* or “*measurements could not be taken on the move”* as reasons for non-regular use were less likely to measure as frequently as instructed. The latter could be included in the assessment of a patient’s suitability for this form of medical care. With regard to measurement frequency, a mean of 20 measurements per month could serve as target reference. However, as qualitative data from both, patients and physicians, show, this should be handled more flexibly and according to individual needs. Furthermore, it is noteworthy that disease duration was a significant negative predictor, whereas age was not. Age did not seem to influence the results, whereas individuals with shorter disease duration may be less adapted to their medical condition and more interested in fulfilling project requirements. This should be investigated in further research.

### Strength and limitations

One strength of this study was the inclusion of interview partners from the different groups of persons involved. To minimize bias due to unconscious assumptions, analyses were conducted independently by two researchers under the supervision of an experienced researcher in the field.

Nevertheless, some limitations must be mentioned. Invitations to participate in the interviews were sent to patients who the study nurse considered suitable to respond which may have caused selection bias. Future studies should also record the stage of the disease and specify more precisely which patients should be included in the interviews. The recruitment process may also have introduced selection bias, which was addressed by explicitly inviting patients who terminated the project prematurely due to dissatisfaction. Still, patients satisfied with the technical applications may have been overrepresented. An even stronger selection bias concerns healthcare providers, who were more reluctant to respond to the telephone interview invitation than previously assumed. Data saturation was reached, as no new major themes emerged from the last interview with a member of the medical staff. However, it is possible that GPs who were less satisfied than the respondents included were not available for an interview, and their perceptions are therefore not represented. Furthermore, the results are based on a single region in Germany and may reflect regional specificities; generalization is therefore limited.

Quantitative results should also be interpreted with caution due to the small sample size. Various determinants of patient satisfaction with the care of their chronic disease using PACIC-SF were based on patient self-assessment. Thus, recall bias may have occurred, particularly if patients have difficulty in accurately assessing their care retrospectively over a longer period of time, as described by others [35]. Furthermore, questions about the degree of satisfaction with medical care and about smoking cessation advice were formulated slightly different at t0 and at t1, which should be taken into account when interpreting these results. Moreover, validated and reliable instruments were used for this study. However, validating them for the study population was not the focus of this study.

## Conclusion

It can be concluded that an important aspect of implementing a digital application for asthma and COPD monitoring is the combination of technical and medical feedback, which may enhance self-efficacy, as observed in the interviews of this study. Patients included in the study were required to have a smartphone and needed to be able to work with these devices. Also, a disease-related education is required. In general, the appropriate measurement frequency should be determined individually by the physician and the patient. Attending physicians should be motivated to give regular personal feedback to the patients, even if the results are inconspicuous from a medical point of view. It can be assumed that time-saving aspects, such as efficient monitoring and fewer practice visits and examinations, could be emphasized when considering the delegation of monitoring tasks to practice staff with already high workloads. Furthermore, measures to improve disease education and vaccination rates are needed and should be the subject of further research.

## Supplementary Information

Below is the link to the electronic supplementary material.

Supplementary Material 1

Supplementary Material 2

## Data Availability

The datasets used and/or analyzed during the current study are available from the corresponding author on reasonable request.

## Abbreviations

β: Regression coefficient
CCM: Chronic Care Model
95% CI: 95% Confidence Interval
COPD: Chronic Obstructive Pulmonary Disease
DMP: Disease Management Program
GP: General practitioner
M: Mean
OR: Odds ratio
PACIC: Patient Assessment of Chronic Illness Care
RCT: Randomized controlled trial
SD: Standard deviation
