# Supplementary material for: Determinants of digital home spirometer use and quality parameters in management of patients with chronic obstructive respiratory disease and asthma in general practice: a mixed methods study
Source: BMC Health Serv Res. 2026 Jun 30;26:887. doi: 10.1186/s12913-026-15048-2 (PMC13317268; doi:10.1186/s12913-026-15048-2)
Supplement: Supplementary file 1 — Supplementary Material 1 [file 12913_2026_15048_MOESM1_ESM.pdf]

## Qualitative part

### Interview guide

| Main questions (used for all participants) |
|---|
| 1) What experiences have you had with using the spirometer/app?<br>3) What challenges did you face when using the spirometer/app?<br>4) What hindered the use of the spirometer/app?<br>5) What would promote the use of the spirometer/app?<br>6) What advantages/disadvantages do you see for patients with this form of care?<br>7) Are there any other aspects you would like to mention? |
| **Questions concerning the telemedical devices** |
| Patients:<br>8a) What has changed for you personally as a result of using the spirometer/app?<br>9a) How has using the spirometer/app affected the way you deal with your illness?<br>10a) How has using the spirometer/app affected your condition? |
| Medical staff:<br>8b) What advantages/disadvantages do you see for yourself/for doctors with this form of care?<br>10b) What challenges did you face when implementing it in everyday practice?<br>11a) What challenges did you face when installing the spirometer/app? (Pneumological assistant)<br>11b) What would speak in favor of or against the practice staff taking over device instruction? (Medical assistant) |

## Quantitative part

## Quantitative questions at t0

### Quality indicators

| |
|---|
| Have you received patient education for your asthma/COPD? |
| Do you smoke? |
| If you smoke, have you received advice on how to quit smoking? |
| Do you get vaccinated against influenza every year since you were diagnosed with asthma/COPD? |

*Note.* Answers given in dichotomous yes/no response options.

### Patient Assessment of Chronic Illness Care (PACIC) Short-Version

| | What percentage of the time was I … |
|---|---|
| **PACIC- Item 1** | …given choices about treatment to think about |
| **PACIC- Item 2** | …satisfied that my care was well organized |
| **PACIC- Item 3*** | … helped to set specific goals for a healthier lifestyle (e.g. healthy nutrition, exercise) |
| **PACIC- Item 4** | …given a copy of my treatment plan |
| **PACIC- Item 5** | … encouraged to get to a specific group or class to help me cope with my chronic condition |
| **PACIC- Item 6*** | …asked questions about my health habits (e.g. whether I smoke) |
| **PACIC- Item 7** | …helped to make a treatment plan that I could carry out in my daily life |
| **PACIC- Item 8** | …helped to plan ahead so I could take care of my condition even in hard times |
| **PACIC- Item 9** | …asked how my chronic conditions affect my life |
| **PACIC- Item 10** | …contacted after a visit to see how things were going |
| **PACIC- Item 11*** | …told how my visits with other types of doctors, (e.g. a referral to an eye doctor), helped my treatment |
| **Additional question** | Overall, how satisfied are you with the medical care you receive for your chronic conditions? |

*Note. Answer options for each item was an e*leven-point percentage scale (10% increments), range "never 0%" to "always 100%".

* Items slightly adapted for this study population

## Quantitative questions at t1

| In retrospect, do you consider your project participation usefull? |
|---|

*Note.* Answers given in dichotomous yes/no response options.

### What were your reasons for using the spirometer? (multiple answers possible)

| | |
|---|---|
| It gave me a feeling of safety. | ❑ |
| To detect a worsening of my symptoms at an early stage. | ❑ |
| To be able to better adjust my medication. | ❑ |
| Because it has improved my understanding of my disease. | ❑ |
| Because the care provided by my family doctor has improved. | ❑ |
| That it was easy to use. | ❑ |

### What were the reasons why you did not perform a measurement? (multiple answers possible)

| | |
|---|---|
| Was not necessary for me because the readings were good. | ❑ |
| A daily measurement was too strenuous for me. | ❑ |
| Measurements could not be taken on the move. | ❑ |
| The frequent measuring made me insecure. | ❑ |
| There have been technical problems. | ❑ |
| Because I did not get any feedback on the readings. | ❑ |

### Quality indicators

| |
|---|
| Have you received patient education for your asthma/COPD? |
| Do you smoke? |
| Did you smoke in the past? |
| If you are a smoker or have smoked in the past, have you received advice on how to quit smoking? |
| Do you get vaccinated against influenza every year? |

*Note.* Answers given in dichotomous yes/no response options.

### Patient satisfaction with chronic care at t1

How have you experienced the treatment of your chronic conditions*? When answering this question, please think back to the last six months. In what percentage of cases have you been satisfied overall with the medical care you have received for your chronic conditions?

In _________ % of the cases

* A chronic condition is an illness that has lasted for at least three months and that affects you physically, mentally or socially.

## Personal information at t0 and t1

| | |
|---|---|
| You are… | ❑ male ❑ female ❑ divers |
| What is the year you were born? | ________________________ |
| Do you live alone? | ❑ Yes ❑ No |
| For how many years have you been diagnosed with asthma/COPD?* | ________________ years |

*only asked at 10
