# Supplementary material for: Determinants of digital home spirometer use and quality parameters in management of patients with chronic obstructive respiratory disease and asthma in general practice: a mixed methods study
Source: BMC Health Serv Res. 2026 Jun 30;26:887. doi: 10.1186/s12913-026-15048-2 (PMC13317268; doi:10.1186/s12913-026-15048-2)
Supplement: Supplementary file 2 — Supplementary Material 2 [file 12913_2026_15048_MOESM2_ESM.pdf]

Supplementary Table 1. Facilitators and barriers to the use of the telemedical applications

	Major category	Subcategory	Quotation
**Facilitators**			
	Sense of obligation		"Disabled? No, it's just that you feel so obligated, that's actually the problem, because sometimes you are not at home or on the road and then you are halfway to bed and you remember "oh, I still have to do this", so those are the moments." (PT18)
	Habituation		"No, I have no idea either, no actually it is like this, you just get used to it, that is all that is new, whether it is glasses or something else, once you have got used to it, it is quite normal." (PT18)
	Visualization of the progression	Symptom control	My state of mind, well, as I said, you have a good feeling because you do it every day, because you have an overview of it and that makes you feel safe." (PT18)
		External control by the doctor	"I think this device is great, especially because I have an overview of my values every day and the doctor is informed at the same time. Before this study, you used to go to the doctor and say "I can't breathe" and now when I come in, my doctor always says: "Oh <name>, I can see that it is always consistently bad." And I think that is great […]" (PT19)
	Self-efficacy	Correlation between condition and findings	"I only took my medication in the morning. Well, I did notice that I felt a bit worse in the mornings as far as air was concerned and during the day regarding the thing with condition when I wasn't feeling well for a week or depending on whether I had an infection or a small touch of a cold, or that was always the worst part of the whole story, but that's how it was and then you always just, yes, I'll say dismissed it t, but now you pay more attention to what you do or you think about what you've done

			wrong or whether you've forgotten something, because I sometimes forget to take my spray in the morning [...]" (PT8)
		Improved self-management	"I think I see it a bit differently at the moment, I accepted it before, and now [thinking noise] I see it a bit differently when I realize it is [thinking noise] deteriorating again that I then have to say [thinking noise] I have to take medication again at some point, then I am more likely to go to the doctor then before..." (PT2)
	Physician contacts	Strengthening of the doctor-patient relationship	"Thanks to this app, I now have more contact with my doctor. We talk about it more often, the doctor calls me more often" (PT2)
		Concept improves GP care compared to pneumological care	"[…] and because I have to go to the lung doctor every six months anyway on the one hand and [thinking noise] he has the values twice, which we always do and when he then does the complete examination once and then that's it, otherwise it's always just that I tell him, because the lung doctor then asks me "how was that, this, this, this and that" and with the GP with the spirometer it's relatively, let's say, more precise and you also get immediate feedback if he sees that the values are falling into the basement or if you're not feeling well for a week or whatever, where you can then react more quickly and much more easily. " (PT8)
	Aspects of remote treatment	Reduction in practice visits/practice examinations	"So I would say three things: it can be used at home, even without the need to go to the doctor's practice, which means I have a kind of controlling of my, or monitoring always sounds so negative, but the possibility of directly taking measurements myself and having them evaluated by the doctor, so there is no need to travel, make an appointment, do the spirometry in the practice itself and then contact the doctor, that is much easier." (PT13 GP)
		Earlier recognition of exacerbations	"So, what I actually think is good is, on the one hand, a certain amount of closer monitoring of the lung function parameters. The fact is that the patients that I have now selected or where I was thinking that they could take part in this project are actually all patients who have very fluctuating lung function disorders that are rather difficult to control and,

			on the one hand, the fact that there is more data available and you can actually take countermeasures a little earlier if you see that the lung function is somehow getting worse [...]" (PT12 GP)
	Integration into the daily practice routine	Asynchronous monitoring	"Apart from that, I have to say that it was quite easy to integrate. The question is always when I can find the time to look at the data, but that's not a problem now, because fortunately you can do it at some point and it is much easier to integrate into a practice plan than anything else." (PT12 GP)
		Access for the entire team	"[...] so I do not think there is much else to complain about, so it is really great that we can all access it, isn't it, that is really great." (PT21 MA)
		Time saving/efficiency	"This overview also saves you time, it is very efficient, time-efficient, because you do not have to look at the individual curves for seven or eight patients, you can see straight away, okay, this one has blown like this (and like this) #0:03:08-3# this is his FVC value or his FEV1, and that is really practical." (PT13 GP)
**Barriers**			
	Understanding the disease	Handling of the disease unchanged	" - Yes - so not so directly, so I have, so I would say now, it has not had such a direct effect. So when I had problems with my lungs, I realized that without a measuring device, you could perhaps see the drop in volume more dramatically or something, but you had to do something anyway and somehow got through it with medication or went to the doctor at some point. Would the spirometer have helped you directly or - I don't know, no." (PT11)
		Insufficient comprehensibility of the measured values	"[...] I would like to know what FEVE means there. Yes. I would, have 29 there, what that means there. Next to it is 81, yes." (PT3)
		Lack of medical feedback	" I'll say if everything is okay, maybe a little text message or a WhatsApp to say it's good. <Name> sent a text message from time to

			time, I have to say, but also from the GP maybe saying "Okay, everything's okay with you", or maybe "if you have any questions, you're welcome to call back", as is always the case." (PT16)
	Dyspnea		"Well, at the moment I'm not doing so well [thinking noise] that's - I have [thinking noise] breathing problems at the moment, I also have quite severe back pain at the moment... so it's not going so well, and then blowing doesn't go well either, so breathing into the spirometer, […]" (PT1)
	Project-related requirements	Forgetting measurements	"Did not prevent anything, I just forgot to do it very often, because I always had it in the kitchen and well, I just had it where I said "then we'll do it tomorrow" and then sometimes I simply postponed it and then I forgot and - yes. So it wasn't because of the device that I didn't do it, but because of me, right?" (PT20)
		Less usage on vacation and at weekends	"[…] so while I always had to go to work in the morning, I always used it before. I have to admit, I always use it a little less at the weekend or when I'm on holiday." (PT7)
	Technical requirements	Newer smartphone required	"It just had to be a newer mobile phone, so what does newer mean [so not actually new], but as a rule it can't be more than seven or eight years old... and that the patients have an e-mail. This is sometimes not the case with patients. So basically, participation fails, if at all, because the patients don't have a suitable smartphone. - But apart from that, there aren't really any challenges." (PT15 MA)
		Internet connection, Bluetooth connection must be activated	"[...] because you still have to connect to your smartphone and then share your location and switch on Bluetooth, which I don't have switched on by default. It's still a bit of a hurdle, where you say, do I actually have the smartphone in the bathroom and do I switch it on, so sometimes the hurdle is really to measure, I've noticed [...] (PT11)

	Presumed age-related difficulties with technology		"It's just that there's a huge difference between 75-year-old country dwellers who, for example, have worked in a job where they had little to do with technology and a city dweller who is 40 years and has basically already grown up with technology. So we realize that very simple things like "I have to connect to the internet" or "I have to download an app" are real hurdles for some people. Of course, this can only be taken away from them to a limited extent, but this is another topic where there are certainly still reserves. (PT13 GP)
	Clinical assessability		"Well, I'm quite an old-school doctor, the disadvantage is always that I don't see the patient. That's just the way it is, especially in family medicine, when you've known people for a long time, and they're sitting opposite me, so I always compare it a bit with a DMP appointment, where I also do a LuFu and where I see the patient and then I simply know within an extremely short time whether they're doing well or badly and what they look like and well, I usually always combine it with a physical examination, which is just naturally missing with such a digital version." (PT12 GP)
	Additional workload	Delegation	" I: […] What challenges did you face when implementing this in everyday practice? PT: [contemplative noise] None really, as I've been doing it on my own up to now, i.e. my girls, I'd still like to delegate it a bit so that they can also get involved, so I don't see a big problem in that respect. I: And what has prevented you from delegating it so far? PT: Quite simply the fact that they have been under constant stress since the beginning of the year, also due to illness, because there are staff absences and there is already a basic defensive attitude, even if I say it's not a big effort. But we will tackle that [laughs]." (PT14 GP)
		Help for instruction/installation from a practice perspective	"So I would see disadvantages if the training had to be done internally. That would be an effort that I think we can not make at the moment, we simply have too many other tasks.That would be a disadvantage, so to speak, it's very fortunate that the study is also financially backed, so to speak, that a pneumological assistant simply takes care of it. [...] but that will be difficult to delegate to the general practices, or I see that as

			difficult. We simply have a great employee in <name>, and she does it very well and that makes it so easy. It's a simple system, if we had to take on this training task, so to speak, that would make the whole thing more difficult again." (PT13 GP)

Note. Abbreviations: FEV1 - forced expiratory volume in the first second; FVC - forced vital capacity value or his FEV1; GP – general practitioner; LuFu – lung function
